# Growing sensitivity of maize to water scarcity under climate change

**DOI:** 10.1038/srep19605

**Published:** 2016-01-25

**Authors:** Qingfeng Meng, Xinping Chen, David B. Lobell, Zhenling Cui, Yi Zhang, Haishun Yang, Fusuo Zhang

**Affiliations:** 1 College of Resources & Environmental Sciences, China Agricultural University, Beijing 100193, China; 2 Department of Earth System Science and Center on Food Security and the Environment, Stanford University, California 94305, USA; 3 Institute of Crop Science, Chinese Academy of Agricultural Sciences and the Key Laboratory of Crop Physiology and Ecology, Ministry of Agriculture, Beijing 100081, China; 4 Chinese Academy of Meteorological Sciences, Beijing 100081, China; 5 Department of Agronomy and Horticulture, University of Nebraska-Lincoln, NE 68583, USA

## Abstract

Climate change can reduce crop yields and thereby threaten food security. The current measures used to adapt to climate change involve avoiding crops yield decrease, however, the limitations of such measures due to water and other resources scarcity have not been well understood. Here, we quantify how the sensitivity of maize to water availability has increased because of the shift toward longer-maturing varieties during last three decades in the Chinese Maize Belt (CMB). We report that modern, longer-maturing varieties have extended the growing period by an average of 8 days and have significantly offset the negative impacts of climate change on yield. However, the sensitivity of maize production to water has increased: maize yield across the CMB was 5% lower with rainfed than with irrigated maize in the 1980s and was 10% lower (and even >20% lower in some areas) in the 2000s because of both warming and the increased requirement for water by the longer-maturing varieties. Of the maize area in China, 40% now fails to receive the precipitation required to attain the full yield potential. Opportunities for water saving in maize systems exist, but water scarcity in China remains a serious problem.

To meet the needs of the global population, which is expected to peak at 8.5–10 billion in 2050, grain production must increase by at least 50% and perhaps by as much as 110% relative to production in 2006^1^,^2^,^3^. Maize is one of the world’s most important cereals, and its production must roughly double to meet the growing demand for food, biofuel, and livestock feed, especially in developing countries^4^. Achieving these increases, however, will be difficult because of water scarcity, which is already a critical problem in many parts of the world and is expected to become more severe in the future^5^. While maize is widely cultivated from the Southern to the Northern Hemisphere, and from arid and semi-arid to humid and semi-humid areas^6^, water scarcity will increasingly constrain maize production. In addition, climate change will likely affect water supply and water demand, heightening this problem in the future^7^,^8^,^9^,^10^. Climate change is also likely to involve warming, which will further compromise maize yields^11^,^12^.

Many studies have suggested that climate-change induced reductions in crop duration and thus yield (higher temperatures reduce the growing period) can be prevented by the planting of varieties that require more time to mature^13^,^14^,^15^. That this suggestion has been followed in some parts of the world and represents a response to climate change has been documented^14^,^16^,^17^. In addition to resulting in increased dry matter accumulation, however, the longer growth period of such modern, longer-maturing varieties also results in a greater water requirement for water. This could increase the susceptibility of yields to water scarcity and adverse weather. In Europe, farmers have adopted shorter-maturing rather than longer-maturing oat varieties in response to climate change because of concerns about late-season drought^18^. Thus, the recent trend toward planting longer-maturing maize varieties in response to increased temperatures could increase the demand for water when the needed water is increasingly unavailable. The relationship between this increased demand for water and water availability in maize production in China and elsewhere has not been quantified.

In this study, we focus on the Chinese Maize Belt (CMB), which in 2012 contributed 78% of the total maize production in China and 18% of the global production^19^,^20^. The goal of this study was to understand how the importance of water for maize yield has evolved with recent (from the 1980s to the 2000s) changes in variety and climate. The CMB (97.5°−135.1° E, 21.1°−53.6° N) covers a wide region of nearly 38 degrees of longitude and 33 degrees of latitude (Fig. 1a,b). It extends from southern tropical and sub-tropical systems (southwest China, SW) at low latitudes to cool-temperate systems (Northeast China, NE) at high latitudes^21^. It also ranges from arid and semi-arid areas (annual precipitation <320 mm) to humid and semi-humid areas (annual precipitation >1000 mm). Because of the diversity of climates across the large range of latitudes, the CMB serves as an excellent laboratory for investigating the effects of varietal and climatic changes on the yield of both rainfed and irrigated maize.

## Results and Discussion

### Climate trends

**Figure 1:**
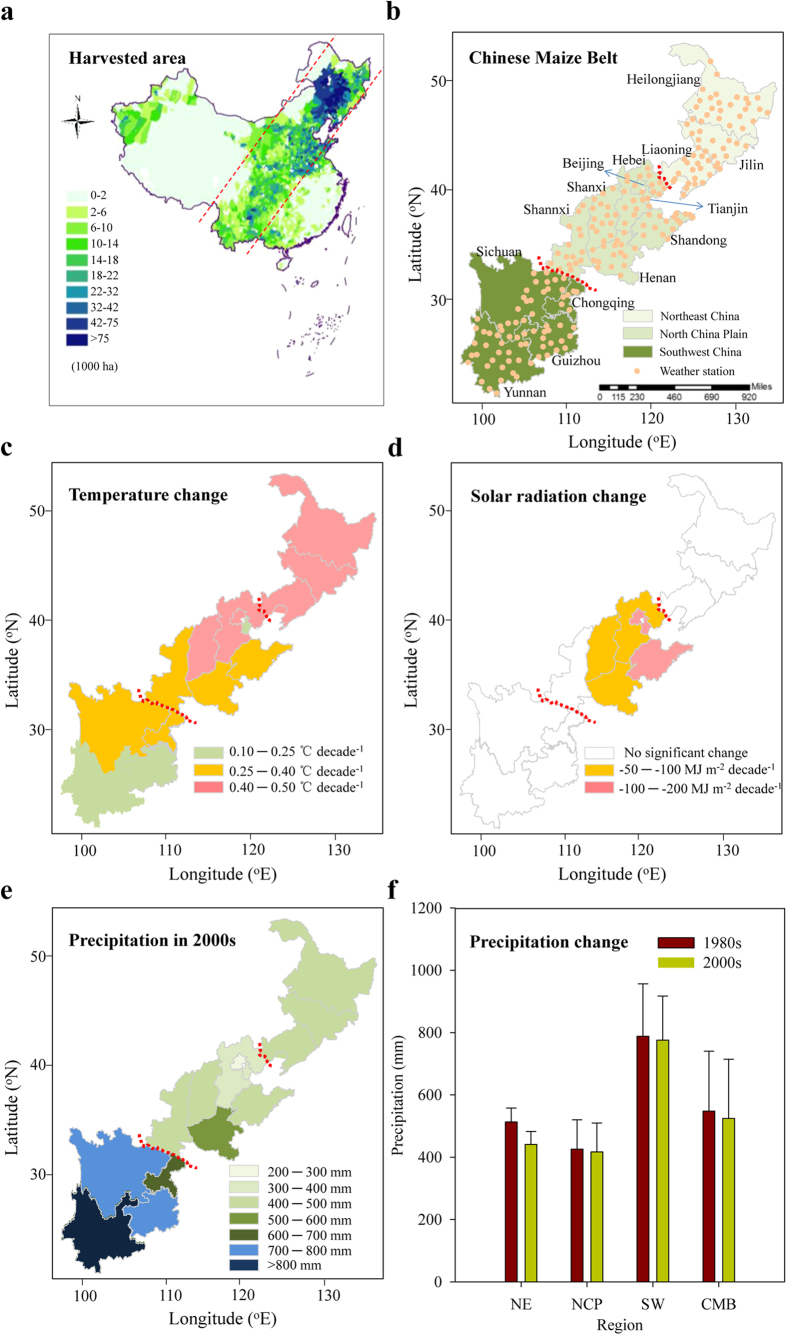
Maize production areas in China, and the Chinese Maize Belt (CMB) and its climates during the maize growing season. (**a**) Maize production area according to county-level data. (**b**) Location of CMB and weather stations. (**c**) Temperature changes (increases in all cases) from 1980–2009. (**d**) Solar radiation change from 1980–2009. (**e**) Average precipitation in the 2000s. (**f**) Average precipitation in regions of the CMB in the 1980s and 2000s. In any region, the precipitation during the maize growing season did not significantly differ between the 1980s vs. the 2000s (*P* < 0.05). NE, NCP, and SW refer to Northeast China, the North China Plain, and Southwest China, respectively. Error bars indicate one standard deviation. We first made the standard module with ArcGIS 10.0 for the province level and then copied to the PowerPoint 2010. Then we made the individual map. Second, the maps in Fig. 1a–f were generated in PowerPoint 2010.

For the entire CMB, average temperatures during the maize growing season increased significantly from 1980–2009 (Supplementary Figure S1); the average temperatures were 21.8 °C from 1980–1989 and 22.6 °C from 2000–2009 (Supplementary Figure S2). The average temperature increase per decade since the 1980s has been 0.37 °C, with faster warming in temperate systems (0.42 °C per decade for the NE and 0.36 °C per decade for the North China Plain, NCP) than in the sub-tropical and tropical systems (0.29 °C per decade for the SW) (Fig. 1c). This warming is substantially higher than the annual temperature increase at the global scale (0.13 °C increase per decade), and is also higher than the increase during the maize growing season in the USA from 1980–2010^12^. Significant decreases in solar radiation were observed mainly for the NCP (Fig. 1d and Supplementary Figure S3). Precipitation during the maize growing season differed greatly among areas, ranging from less than 300 mm in some areas of the NCP to more than 1000 mm in some areas of the SW; precipitation did not change, however, in any area from the 1980s to the 2000s (Fig. 1e,f and Supplementary Figure S4). From 1980–2009 in the maize season, the precipitation in the NE, NCP and SW averaged 491, 413, and 786 mm, respectively.

### Effects of climate change and variety change on maize yield

**Figure 2:**
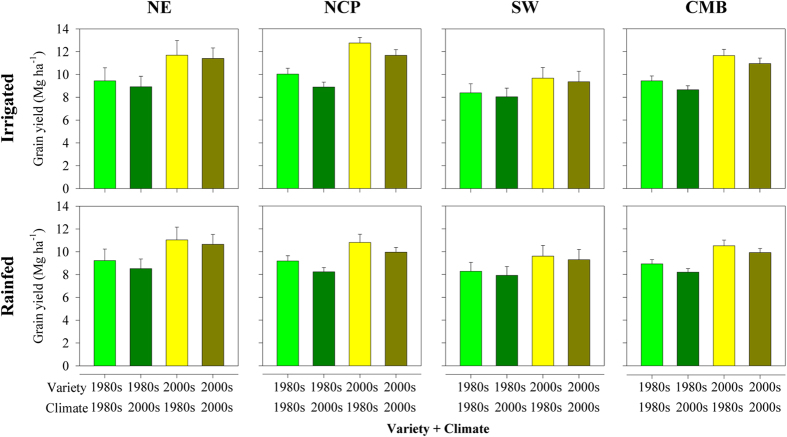
Simulated combined effects of variety (varieties planted in the 1980s vs. 2000s) and climate (climate in the 1980s vs. 2000s) on grain yield in rainfed maize and in irrigated maize for the entire Chinese Maize Belt (CMB) and for three regions (NE, NCP, and SW) in the CMB. NE, NCP, and SW refer to Northeast China, the North China Plain, and Southwest China, respectively. Error bars indicate one standard deviation.

Simulating with the Hybrid-Maize model^22^,^23^, maize yield of the 1980s variety for the entire CMB decreased by an average of 8% due to climate change from the 1980s to the 2000s for both irrigated maize (from 9.5–8.7 Mg ha^−1^) and rainfed maize (from 8.9–8.2 Mg ha^−1^) ((grain yield of 1980s’ varieties*1980s’ climates - grain yield of 1980s’ varieties*2000s’ climates)/grain yield of 1980s’ varieties*1980s’ climates) (Fig. 2). This decrease was greater than the 3.8% decrease in global maize yield attributed to climate over the same period^12^. Simulated yields with varieties used in the 1980s decreased by 10–11% in the NCP but by only 5–8% in the cold-temperate maize systems in the NE. For tropical and sub-tropical maize in SW China, grain yield has decreased 4% since the 1980s. The only area without negative effects of climate change (1980s vs. 2000s) was Heilongjiang Province (Supplementary Figure S5), which is located in northernmost region of China where the baseline temperature in the 1980s was not above the optimum for maximum grain yield^24^. For all regions, the scenarios analysis for irrigated maize showed the attribution of maximum and minimum temperature for yield decrease from the 1980s to the 2000s was similar (Supplementary Table S1). For NCP, the 4% yield decrease for irrigated maize from the 1980s to the 2000s was attributed to the decrease in solar radiation.

To understand how farmers changed their crop management in response to warming since the 1980s, we collected information on sowing date and varieties. Sowing date was similar in the 1980s (average DOY = 135) and the 2000s (average DOY = 133) (Supplementary Figure S6). Regarding varieties, those planted in the 2000s had significantly more total growth days (8 days more) than those planted in the 1980s. Of the additional growth days, seven occurred in the post-flowering reproductive stage. The total growing degree-days (GDD, ≥10 °C) were therefore 12% greater for varieties in the 2000s than for those used in the 1980s (1403 vs. 1575 GDD). The more recent varieties thus have more time for grain filling and use light, heat, and other resources more efficiently than the varieties used in the 1980s.

The yield performance was compared for combinations of two kinds of varieties (those planted in the 1980s and the 2000s) with two climate periods (the 1980s and the 2000s) (Fig. 2). For the entire CMB area, average grain yield increased by 11–16% (from 9.4–10.9 Mg ha^−1^ for irrigated maize and from 8.9–9.9 Mg ha^−1^ for rainfed maize) due to farmers switching to longer-maturing varieties from the 1980s to the 2000s ((grain yield of 2000s’ varieties*2000s’ climates - grain yield of 1980s’ varieties*1980s’ climates)/grain yield of 1980s’ varieties*1980s’ climates). Observed maize yield increased 34% for the CMB between the 1980s and the 2000s^20^. This indicated that benefits from changing varieties represented 32–47% of the overall yield gains over this period. Similarly, 50–60% of maize yield increase was attributed to variety improvement (genetic improvement) in the USA^25^. Among different regions, the highest yield increase was observed in the NE, where yield for the varieties used in the 2000s with the climate from the 2000s was 21% higher for irrigated maize (and 15% higher with rainfed maize) than with varieties used in the 1980s and with the climate from the 1980s. With the same comparisons of varieties and climate in SW China, the grain yield increased by 10% of for both water conditions. In the NCP, the grain yield increased as much as 16% for irrigated maize but by only 8% for rainfed maize. In most regions, the estimated effects of climate trends on new varieties were negative and with similar magnitude as effects on older varieties. This suggests that while the new varieties have helped offset climate-related losses, their yields would have been even higher without climate change.

### Growing sensitivity of maize production to water

**Figure 3:**
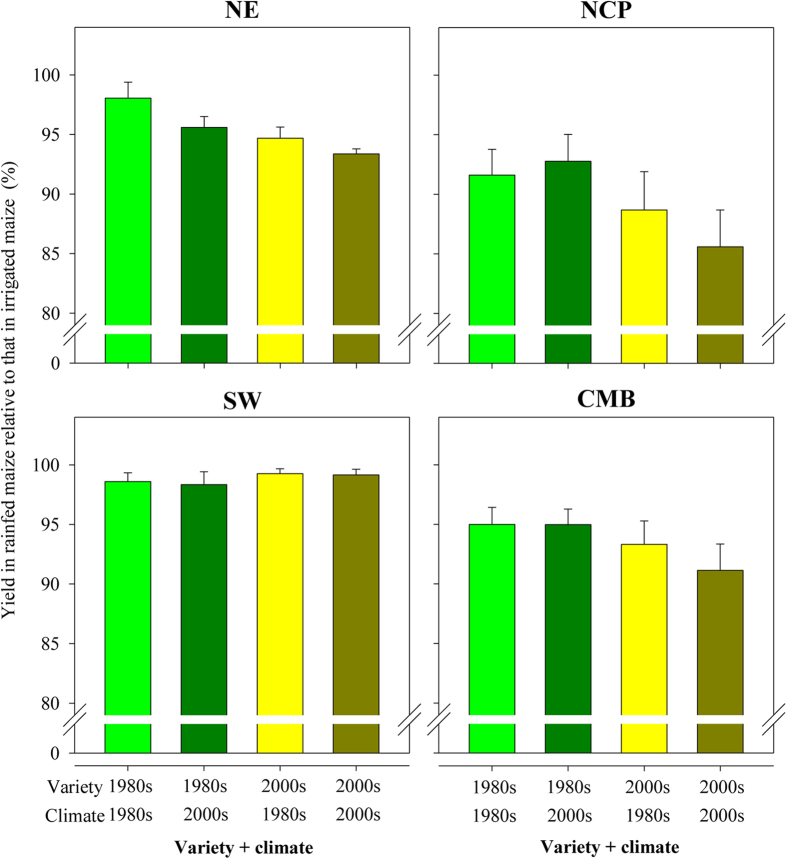
Simulated grain yield in rainfed maize expressed as a percentage of that in irrigated maize combinations of two kinds of varieties (those planted in the 1980s and 2000s) and for two climates (the 1980s and 2000s) for the entire Chinese Maize Belt (CMB) and for three regions (NE, NCP, and SW) in the CMB. NE, NCP, and SW refer to Northeast China, the North China Plain, and Southwest China, respectively. Error bars indicate one standard deviation.

To determine whether the gap between irrigated and rainfed maize yields in the region is increasing, rainfed yield was expressed as a percentage of irrigated yield for all combinations of two kinds of varieties and two climate periods (Fig. 3). For the entire CMB, yield was 5% lower for rainfed maize than for irrigated maize when varieties used in the 1980s were combined with climates from either the 1980s or the 2000s. For the entire CMB, yield was 10% lower when varieties used in the 2000s were combined with the climate from the 2000s. The increased difference in rainfed vs. irrigated maize mostly occurred in the NE and NCP, while water remained a non-limiting factor in SW China. The importance of climate in causing the drop in rainfed vs. irrigated yields was most apparent in the NE (Fig. 3 and Supplementary Figure S7), which is partly related to slight reductions in precipitation (Fig. 1f) and to increased evaporative demand in a warmer climate^26^.

**Figure 4:**
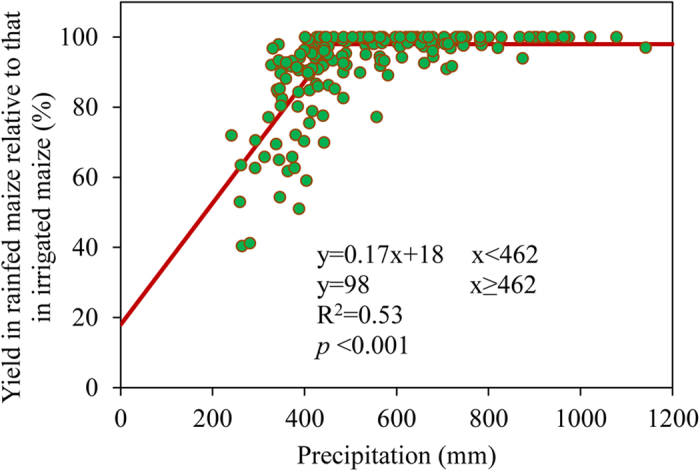
Grain yield in rainfed maize expressed as a percentage of grain yield in irrigated maize plotted against precipitation during the maize growing season for the entire Chinese Maize Belt. The data are based on varieties used in the 2000s and on current precipitation data from local weather stations.

To meet yield potential without irrigation, we found that the critical level of precipitation was 462 mm during the maize growing season for the modern, longer-maturing varieties in the CMB (Fig. 4). Among regions, the NE and NCP required similar levels of precipitation (446–460 mm) to fully achieve the yield potential whereas lower levels of precipitation did not affect yields in the SW (Supplementary Figure S8). Current precipitation in 40% of the entire CMB failed to meet the demand of the modern varieties. Precipitation in 62% of the NE and 60% of the NCP failed to meet the demand of modern varieties. For example, precipitation during the maize growing season in Hebei, Shannxi and Shanxi Provinces in the NCP was only about 350 mm, resulting in a 100-mm gap between maize water demand and supply. This gap substantially decreases the possibility that yield potential will be achieved without irrigation.

The importance of water as a limiting factor is increasing because of the shift to longer-maturing varieties, and because of the adoption of other agronomic measures that have been used to increase yield. In the Midwest of the USA, for example, farmers have increased planting density, which obviously increases the demand for water^27^. Because the CMB encompasses regions with diverse climates, research from the CMB concerning the effects of changes in varieties and climates on maize yields is relevant to many other parts of the world. In many developing countries in southern Asia, Africa,the Middle East, and South America, precipitation in the maize growing season ranges from 300–500 mm, which is near or below the level required to obtain the maize yield potential^6^,^7^; maize yield in these areas is severely limited by water^21^.

As water resources become increasingly scarce, especially under climate change, securing food supplies will depend on increases in water use efficiency^28^,^29^,^30^. Water use efficiency in maize systems can be increased by optimizing irrigation (e.g., by deficit irrigation) and by increasing soil water retention via conservation agriculture, minimum tillage, and the covering of the soil surface in the field (with crop residues or plastic)^28^,^30^,^31^. Water-use efficiency can also be increased by rotations that include crops that are especially efficient in their use of water^32^. Another important approach is to develop high-yielding, stress-tolerant maize varieties^1^,^33^.

In summary, we found that the gap between rainfed yields and irrigated yields substantially increased from 5% in the 1980s to 10% in the 2000s and that 40% of the maize production area in CMB now fails to receive the precipitation required to achieve full yield potential. Growing sensitivity of maize production to water scarcity resulting from a shift to longer-maturing varieties makes adaptation to climate change especially difficult and suggests that new adaptation measures are needed. These measures include new approaches to agronomic management and water management and the breeding of new varieties.

## Methods

### Study area and weather data

The study area included 14 provinces in the CMB. Weather data were obtained from 216 observational stations of the National Meteorological Networks of Central China Meteorological Agency^34^. These stations were chosen because they followed a south-to-north transect from low and middle latitudes in SW China to high latitudes in NE China (Fig. 1b). Spring maize was the dominant maize system in the NE (Heilongjiang, Jilin and Liaoning Provinces), and the SW (Yunan, Guizhou, Chongqing, and Sichuan Provinces) while summer maize was the dominant system in the NCP (Hebei, Henan, Shandong, Beijing and Tianjin Provinces). In Shanxi and Shannxi Provinces in the NCP, maize was mainly sown in spring.

### Data collection

The 216 weather stations provided daily records of sunshine hours; minimum, mean and maximum temperatures; precipitation; and wind speed from 1980–2009^34^. Daily solar radiation was estimated using a previously published equation^35^.

To assess how farmers have changed to longer-maturity varieties, we collected data on maize phenology (sowing, silking, and maturity dates) from both the 1980s and the 2000s from Agrometeorological Experimental Stations for each province in the CMB. Fifty agronomists at 50 experimental stations in the National Maize Production System in China were surveyed to verify the phenological information. The sowing, silking, and maturity dates for each province for varieties planted in the the 1980s and the 2000s are shown in Supplementary Table S2. The parameter growing degree-days (GDD, ≥10 °C) was used to characterize the varieties and for model simulation. At each station in both the 1980s and the 2000s, the GDD is the average of all collected varieties at that time. According to the average phenological data, the total GDD, pre-, and post-silking GDDs for varieties from both the 1980s and 2000s were calculated based on the average temperature in each province^36^. Total, pre-, and post-silking GDDs for each province for varieties used in the 1980s and 2000s are shown in Supplementary Table S3. For each province, an average GDD was used for crop model simulation.

The soil data used in our study included soil texture, bulk density, initial soil moisture status, coverage of residues, and maximum root depth. These data were also obtained from the Agrometeorological Experimental Stations and the China Soil Scientific Database.

### Crop modeling and simulation

Translating climate trends into potential yield effects requires models. The Hybrid-Maize model was developed by the University of Nebraska-Lincoln in the USA by combining the strengths of the existing specific models represented by CERES-Maize with organ growth and respiration functions from assimilation-driven generic crop models such as SUCROS and WOFOST^22^,^23^. The Hybrid-Maize model can simulate maize yield under both irrigated and rainfed conditions. It can also simulate maize daily development and growth with minimal possible stress. Previous studies have shown that the model performed well in a variety of regions in China^24^. In this study, we relied on the calibrated model from prior work^24^.

To simulate grain yield, the Hybrid-Maize model requires input for daily total solar radiation, maximum and minimum temperature, and evapotranspiration. Other model inputs include each variety’s GDD (GDD at silking and maturity), date of planting, and plant population density. In this study, the real sowing dates for varieties used in the 1980s and 2000s varieties (Supplementary Table S2) and plant population densities (60,000 plants ha^−1^ at all stations) were used in the simulation for both rainfed and irrigated maize with varieties from both the 1980s and 2000s. The change in atmospheric CO_2_ level since the 1980s has not been taken into account in the simulation because the Hybrid-Maize model does not consider CO_2_ changes.

We used Hybrid-Maize model to relate past yield outcomes to weather realizations under a scenario in which varieties were constant, i.e., the same varieties used by farmers in the 1980s were used throughout the simulation; in other words, there was no adaptation to climate change in terms of variety (Supplementary Table S2). Similar simulations were also conducted for the varieties used by farmers in the 2000s. For the model simulation, grain yield with the climate of the 1980s or the 2000s was the average of the 10-year simulation with weather data from 1980–1989 or from 2000–2009, respectively.

### Data analysis

For the entire CMB, the following variables were analyzed with regression to estimate linear time trends from 1980–2009: mean temperature, precipitation, and solar radiation during the maize growing season for each year and each province. Linear time trends were also computed for the simulated grain yield from 1980–2009 based on varieties used in the 1980s. Students’ *t*-tests with 95% or 99% confidence levels were used to evaluate the slope of the linear regression line against time. If a linear regression was significant, we used the average change from the 1980s to the 2000s to describe the overall trend. For the CMB as a whole, the climatic trends for climate and simulated maize yields were calculated as averages based on the area of each province. The relationship between rainfed maize yield as a percentage of irrigated maize yield and precipitation during the maize growing season was evaluated with the linear plateau model in SAS^37^.

## Additional Information

**How to cite this article**: Meng, Q. *et al.* Growing sensitivity of maize to water scarcity under climate change. *Sci. Rep.* **6**, 19605; doi: 10.1038/srep19605 (2016).

## Supplementary Material

Supplementary Information
