# Supplementary material for: Growing sensitivity of maize to water scarcity under climate change
Source: Sci Rep. 2016 Jan 25;6:19605. doi: 10.1038/srep19605 (PMC4726359; doi:10.1038/srep19605)
Supplement: Supplementary Information [file srep19605-s1.pdf]

Supplementary information

Growing sensitivity of maize to water scarcity under climate change

Qingfeng Meng[1,2,3], Xinping Chen[1], David B. Lobell[2], Zhenling Cui[1], Yi Zhang[4], Haishun Yang[5] & Fusuo Zhang[1]

[1] China Agricultural University, Beijing 100193, China.

[2] Department of Environmental Earth System Science and Center on Food Security and the Environment, Stanford University, California 94305, USA.

[3] Institute of Crop Science, Chinese Academy of Agricultural Sciences and the Key Laboratory of Crop Physiology and Ecology, Ministry of Agriculture, Beijing 100081, China.

[4] Chinese Academy of Meteorological Sciences, Beijing 100081, China.

[5] Department of Agronomy and Horticulture, University of Nebraska-Lincoln, NE 68583, USA.

Correspondence and requests for materials should be addressed to X.C. (email: chenxp@cau.edu.cn)

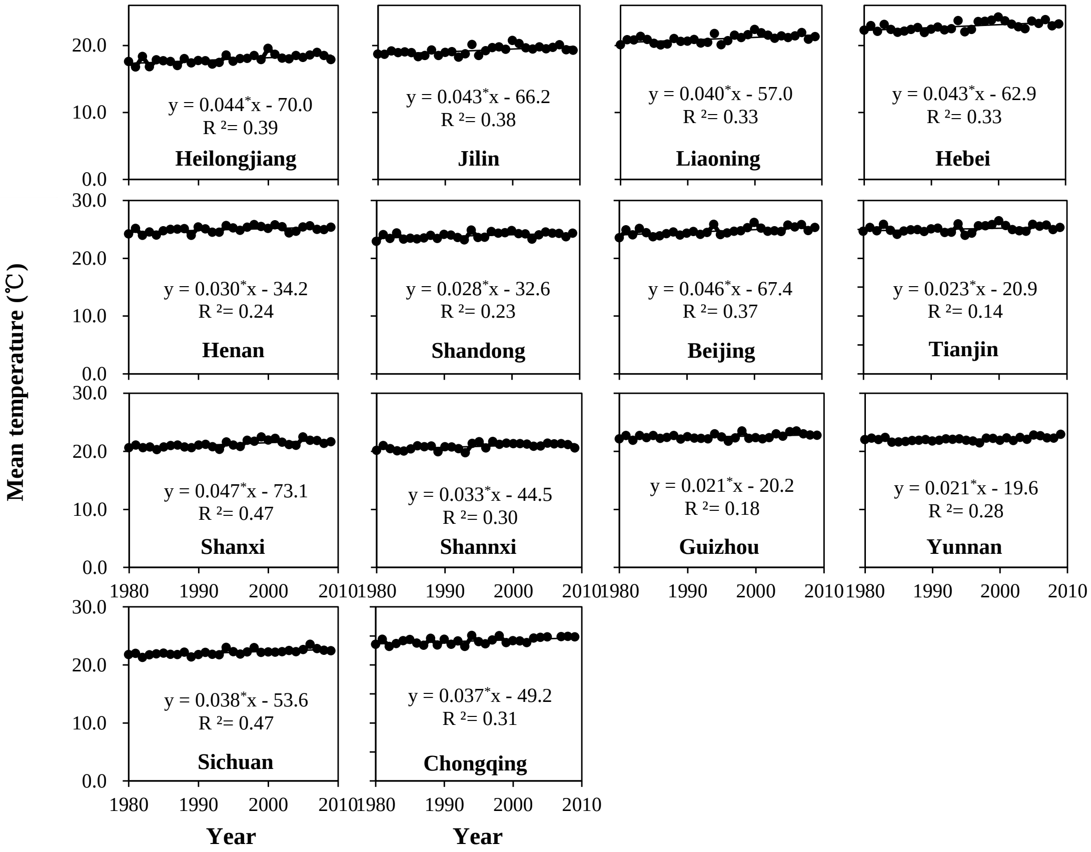

Supplementary Figure S1. The mean temperature change from 1980 to 2009 for each province in Chinese Maize Belt. *P<0.05.

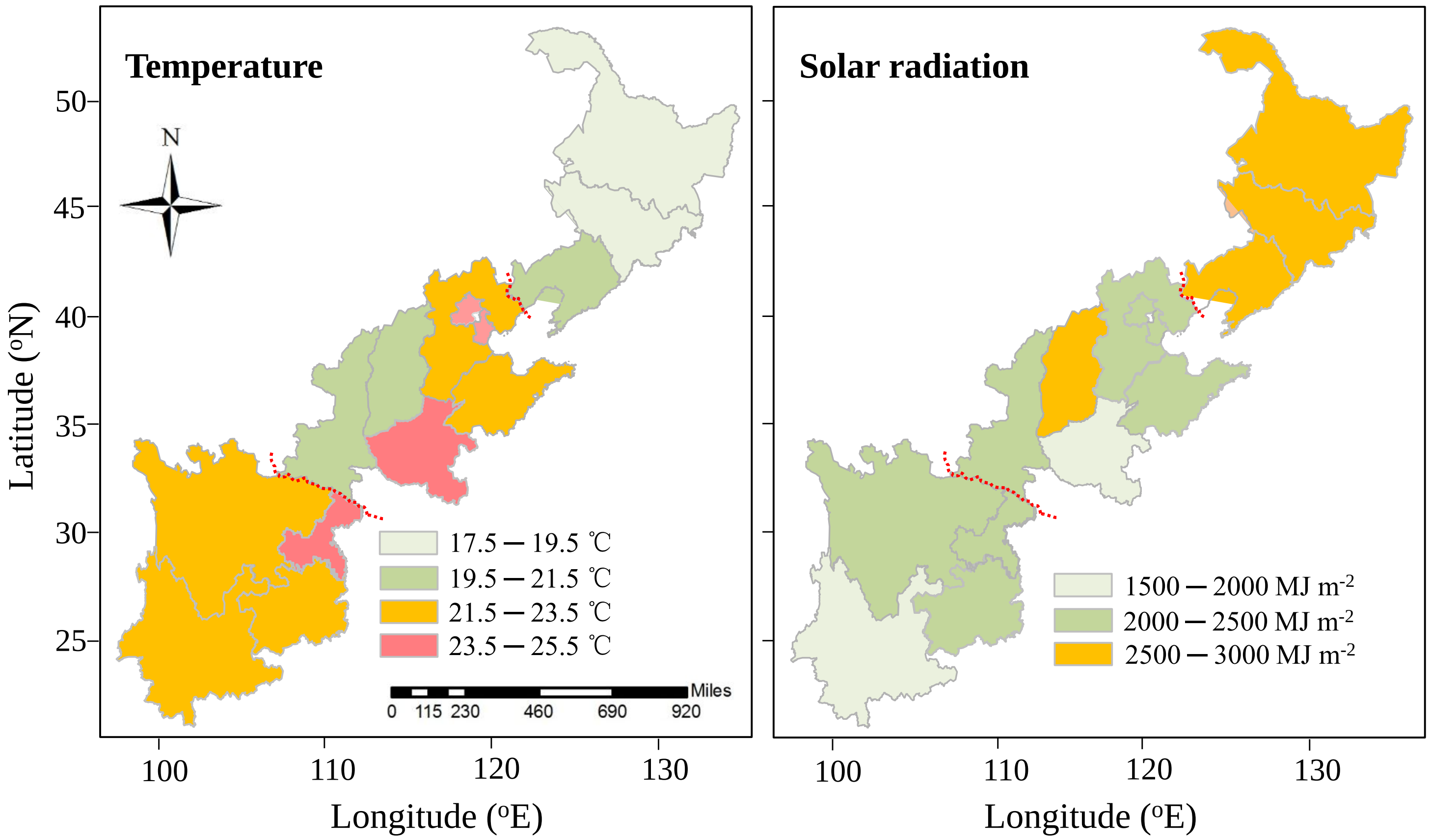

Supplementary Figure S2. The baseline temperature and solar radiation in 1980s (1980-1989) in Chinese Maize Belt. We firstly made the standard module with ArcGIS 10.0 for the province level and then copied to the PowerPoint 2010. Then we made the individual map. Secondly, the maps were generated in PowerPoint 2010.

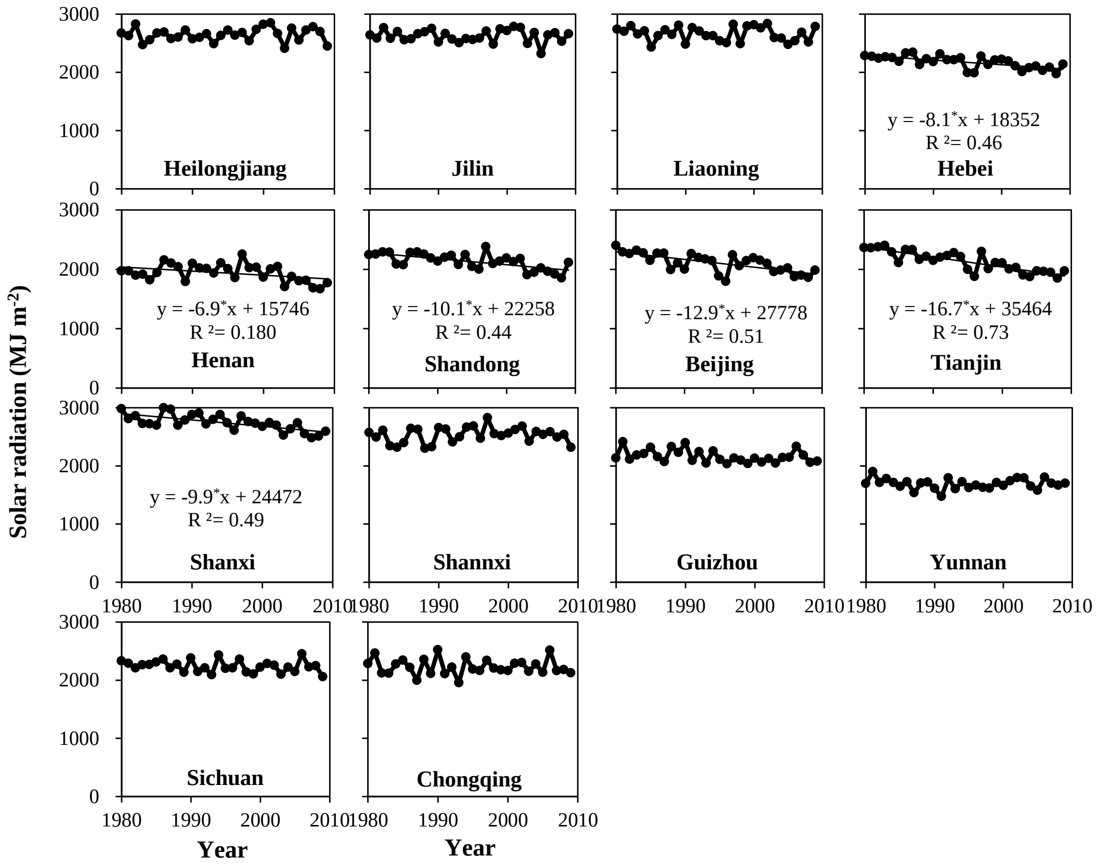

Supplementary Figure S3. The solar radiation change from 1980 to 2009 for each province in Chinese Maize Belt. ***P*<0.05.

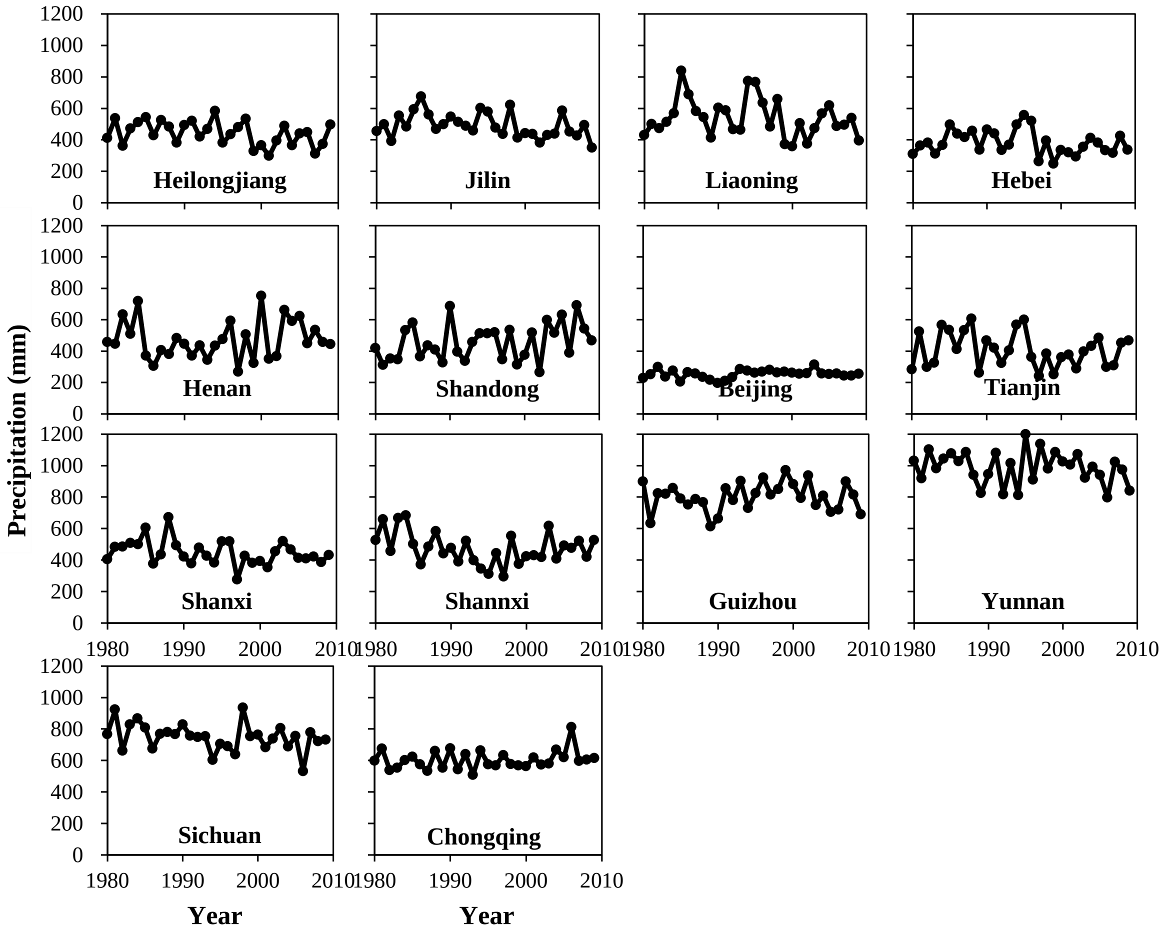

Supplementary Figure S4. The precipitation change from 1980 to 2009 for each province in Chinese Maize Belt.

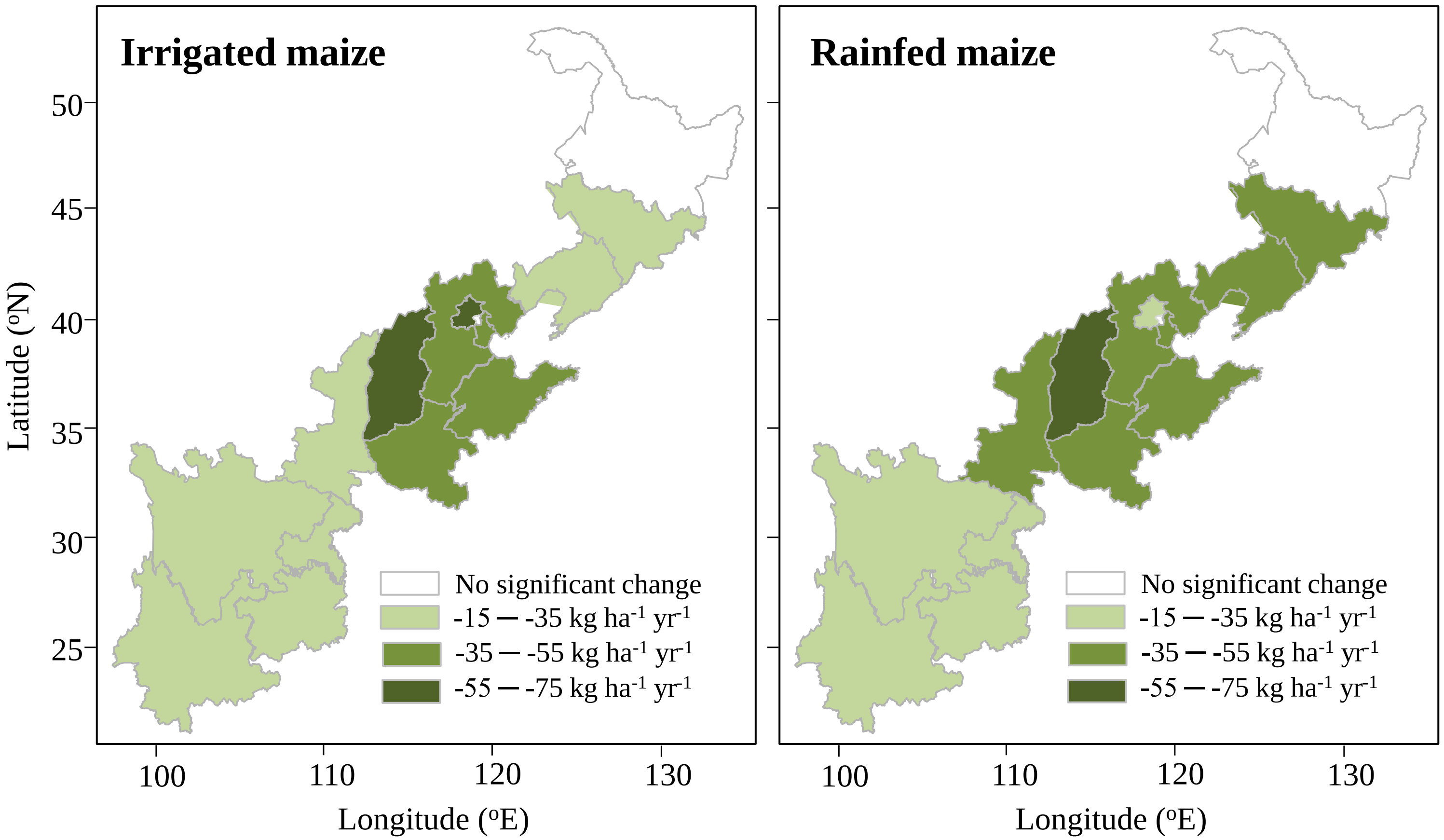

Supplementary Figure S5. Impacts of climate trend on maize yield for irrigated and rainfed maize from 1980 to 2009 with 1980s varieties. We firstly made the standard module with ArcGIS 10.0 for the province level and then copied to the PowerPoint 2010. Then we made the individual map. Secondly, the maps were generated in PowerPoint 2010.

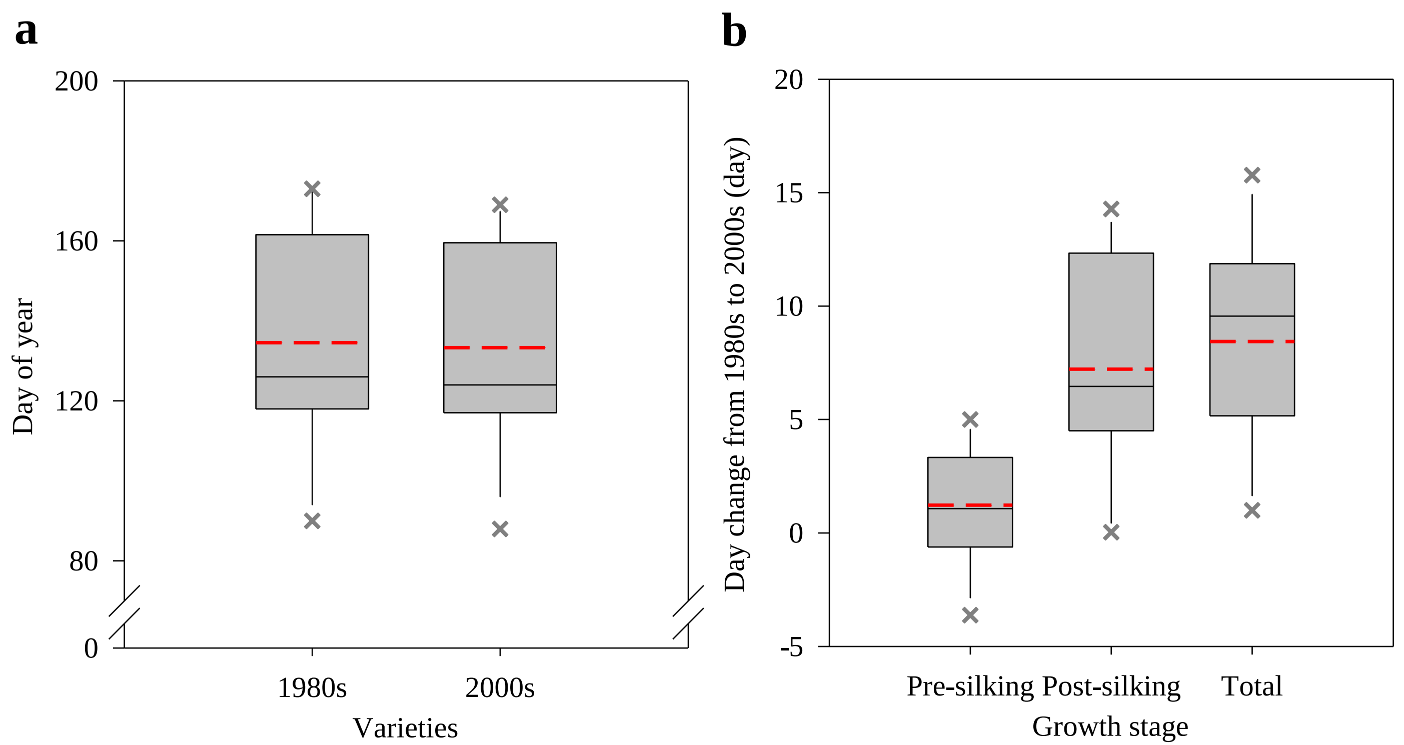

Supplementary Figure S6. Sowing date (a) and growth stages changes (b) between 1980s and 2000s varieties in pre-, and post- silking and total for whole Chinese Maize Belt. Solid and dashed lines indicate median and mean values, respectively. Box boundaries indicate upper and lower quartiles; the line upper and lower indicate 90th and 10th percentiles; cross indicate 95th and 5th percentiles.

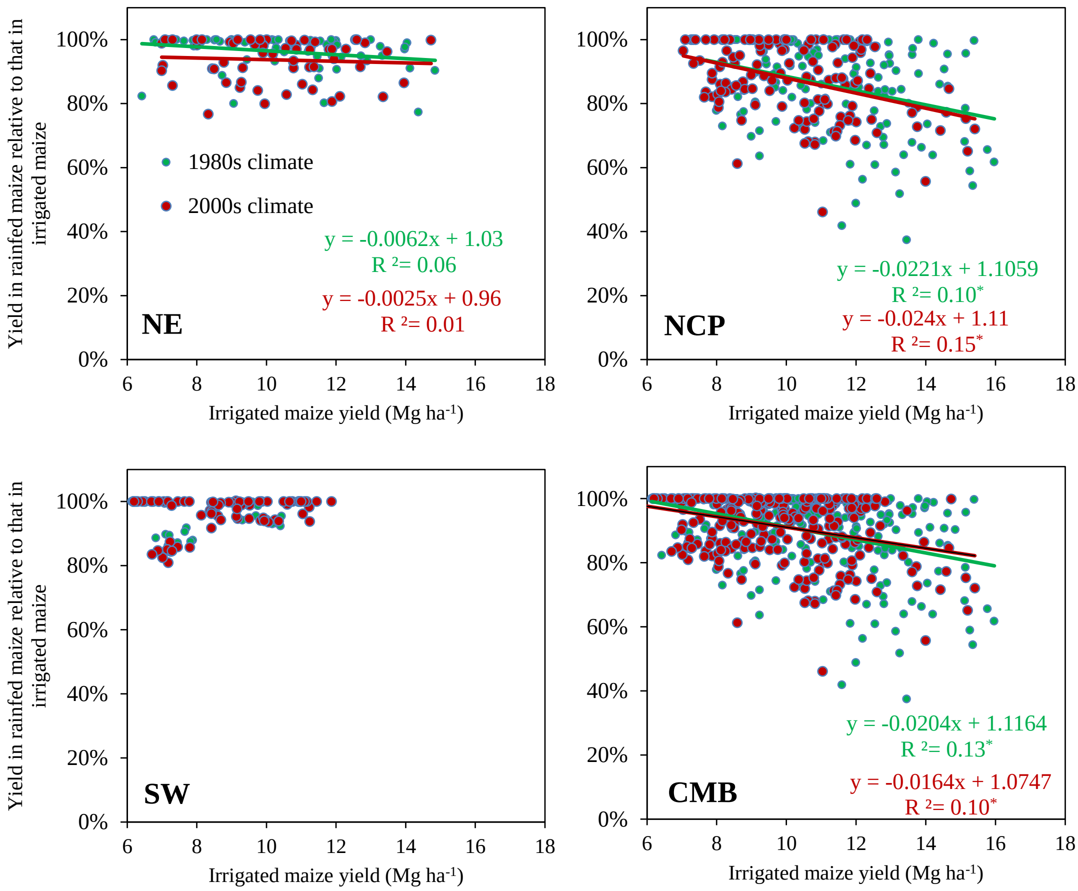

Supplementary Figure S7. Grain yield in rainfed maize expressed as a percentage of grain yield in irrigated maize plotted against irrigated maize yield with the 1980s and the 2000s climates. NE, NCP, SW and CMB is Northeast China, North China Plain, Southwest China and Chinese Maize Belt, respectively.

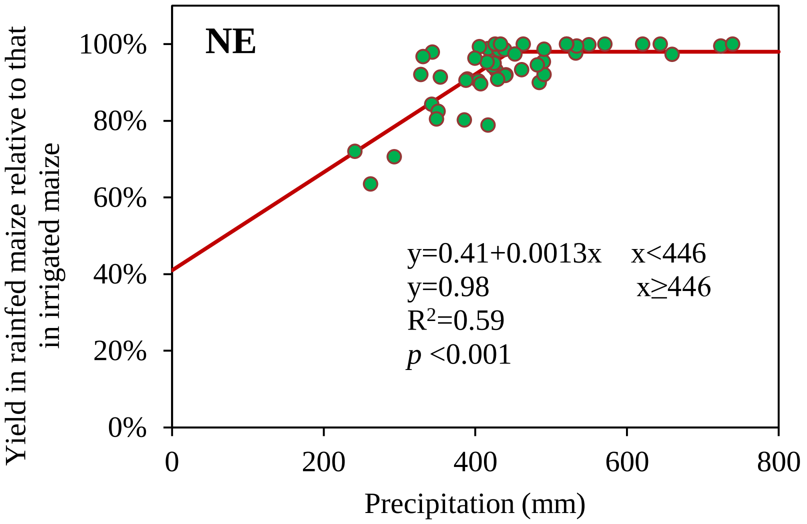

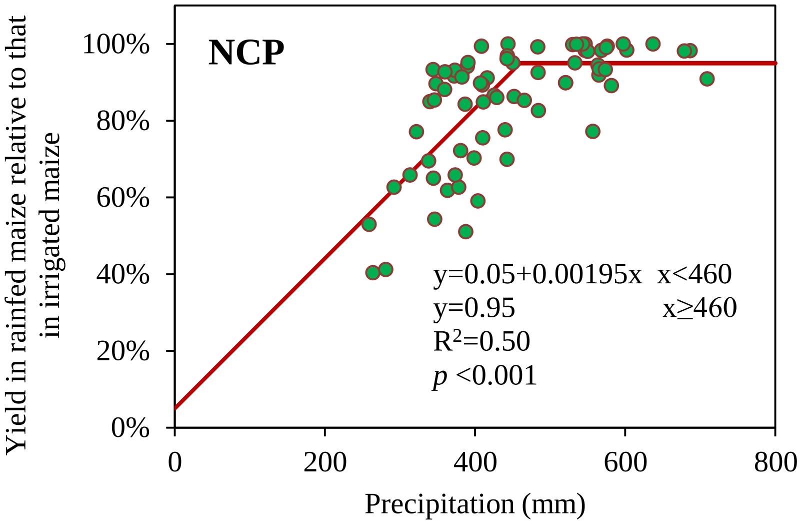

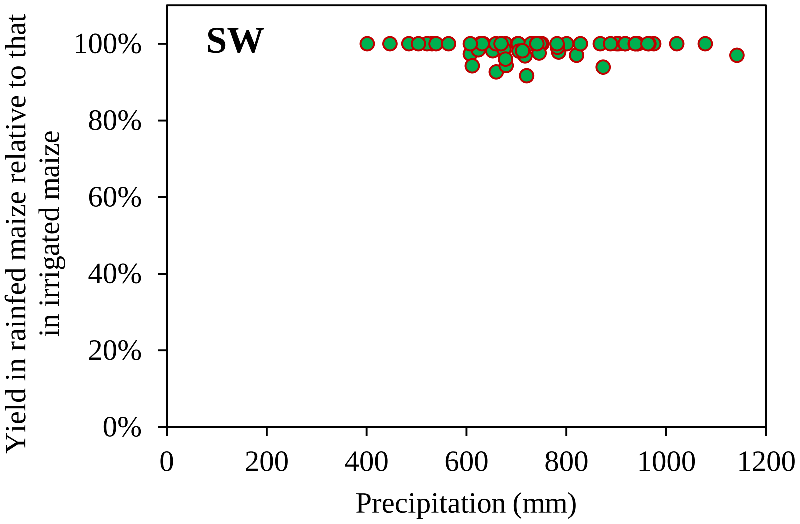

Supplementary Figure S8. Grain yield in rainfed maize expressed as a percentage of grain yield in irrigated maize plotted against precipitation

during the maize growing season. NE, NCP and SW is Northeast China, North China Plain, and Southwest China, respectively.

Supplementary Table S1. Simulated grain yield changes for irrigated maize for different climate variables (minimum temperature, maximum temperature and solar radiation) from 1980s to 2000s with 1980s varieties.

Region	Yield in 1980s	Yield change with varying climate variables from 1980s to 2000s		
		Minimum temperature	Maximum temperature	Solar radiation
	Mg ha^{-1}	--------------------------%--------------------		
NE China	9.5	-2	-3	0
NCP	10.0	-3	-4	-4
SW China	8.4	-1	-3	0
CMB	9.4	-2	-3	-2

Supplementary Table S2. The phenology (sowing, silking and maturity dates) information for each province for both 1980s and 2000s varieties.

Province	1980s variety			2000s variety		
	Sowing	Silking	Harvest	Sowing	Silking	Harvest
Heilongjiang	6-May	30-Jul	16-Sep	4-May	30-Jul	23-Sep
Jilin	29-Apr	27-Jul	15-Sep	30-Apr	28-Jul	21-Sep
Liaoning	27-Apr	26-Jul	13-Sep	28-Apr	26-Jul	18-Sep
Hebei	14-Jun	9-Aug	19-Sep	12-Jun	10-Aug	29-Sep
Henan	9-Jun	6-Aug	14-Sep	7-Jun	2-Aug	17-Sep
Shandong	9-Jun	8-Aug	16-Sep	6-Jun	6-Aug	26-Sep
Beijing	21-Jun	11-Aug	20-Sep	15-Jun	8-Aug	25-Sep
Tianjin	23-Jun	13-Aug	15-Sep	19-Jun	10-Aug	26-Sep
Shanxi	30-Apr	17-Jul	15-Sep	26-Apr	17-Jul	27-Sep
Shannxi	1-May	16-Jul	6-Sep	30-Apr	11-Jul	15-Sep
Guizhou	10-Apr	27-Jun	10-Aug	18-Apr	6-Jul	24-Aug
Yunnan	10-May	10-Jul	7-Sep	10-May	15-Jul	13-Sep
Sichuan and Chongqing	30-Mar	26-Jun	6-Aug	28-Mar	24-Jun	9-Aug

Supplementary Table S3. The growing degree-days (GDD) for each Province for both 1980s and 2000s varieties.

Province	1980s variety			2000s variety		
	Pre-silking	Post-silking	Total	Pre-silking	Post-silking	Total
Heilongjiang	710	430	1140	789	478	1267
Jilin	785	528	1314	887	577	1464
Liaoning	906	617	1523	983	694	1677
Hebei	835	541	1376	958	654	1611
Henan	968	567	1535	977	682	1659
Shandong	892	535	1427	931	696	1627
Beijing	841	519	1360	910	650	1560
Tianjin	878	472	1350	909	660	1569
Shanxi	828	693	1521	949	820	1768
Shannxi	724	606	1330	759	768	1527
Guizhou	852	673	1525	950	748	1698
Yunnan	752	707	1459	804	740	1544
Sichuan and Chongqing	816	563	1379	833	667	1500
